# The efficacy of different types of cerebral embolic protection device during transcatheter aortic valve implantation: a meta-analysis

**DOI:** 10.3389/fcvm.2024.1205943

**Published:** 2024-02-23

**Authors:** Chao Wang, Jingjun Han, Liuyi Lu, Junxiong Qiu, Yuan Fu, Junmeng Zheng

**Affiliations:** ^1^ Department of Cardiovascular Surgery, Sun Yat-Sen Memorial Hospital, Sun Yat-Sen University, Guangzhou, China; ^2^ Department of Thoracic and Cardiac Surgery, The Eighth Affiliated Hospital, Sun Yat-Sen University, Shenzhen, China

**Keywords:** CEPD, TAVI, TAVR, Stroke, Meta-analysis

## Abstract

**Aims:**

Perioperative stroke remains a devastating complication after transcatheter aortic valve implantation (TAVI), and using a cerebral embolic protection device (CEPD) during TAVI may reduce the occurrence of stroke according to some studies. Therefore, we conducted this meta-analysis to determine whether CEPD should be routinely used during TAVI.

**Methods and results:**

The inclusion criteria for this study were randomized controlled trials (RCTs) that examined the outcome of stroke with or without CEPD during TAVI, with a minimum follow-up period of 30 days. The primary endpoint was the occurrence of stroke (including both cerebrovascular accidents and death due to cerebrovascular accidents). The risk of stroke was lower in the CEPD group: RR 0.68, 95% CI 0.49–0.96, *p* = 0.03, *I*^2 ^= 0%. A subgroup analysis was conducted according to the type of CEPD. The risk of stroke was lower in the I&LCCA (filter cover the innominate and the left common carotid arteries) type CEPD group: RR 0.66, 95% CI 0.49–0.96, *p* = 0.03, *I*^2 ^= 36%. However, there was no statistically significant difference in the risk of stroke in the TMCA [filter cover the three major cerebral arteries (innominate, left common carotid, and subclavian arteries)] type CEPD group: RR 0.81, 95% CI 0.36–1.80, *p* = 0.60, *I*^2 ^= 0%.

**Conclusions:**

In this meta-analysis, the I&LCCA-type CEPD can reduce the risk of stroke within 30 days following TAVI, but the TMCA type cannot.

## 1 Introduction

Transcatheter aortic valve implantation (TAVI) was initially introduced in 2002 for the treatment of severe aortic stenosis (1). Over time, its utilization has extended beyond high-risk patients and may now also be used for low-risk patients.

Although the technology of TAVI equipment, the experience of operators, and the use of antithrombotic drugs had all been greatly improved, the incidence of stroke after TAVI was not significantly reduced (2, 3), and studies have shown that it was as high as 2%–5% (4, 5). Attention was drawn to the need to reduce the risk of stroke during TAVI, leading to the development of the cerebral embolic protection device (CEPD).

According to the operating principle of CEPD, using CEPD during TAVI can prevent the entry of various emboli (such as thrombus, vascular fragments, heart tissue) into the cerebral arteries, thereby reducing the incidence of stroke (6–8).

Published randomized controlled trials (RCTs) and retrospective cohort studies were insufficient to demonstrate that CEPD avoids or lowers the incidence of stroke, death, and other complications in patients after TAVI. In addition, the published meta-analysis may not draw firm conclusions due to the insufficient sample size and short follow-up time (4, 5, 9, 10).

A recent RCT enrolled 3,000 patients, which has the potential to provide valuable insights into the true efficacy of CEPD (11). Therefore, we conducted this updated meta-analysis to examine the efficacy of CEPD during TAVI. In addition, we also evaluated different types of CEPD.

## 2 Methods

This meta-analysis adhered to the guidelines established by the Preferred Reporting Items for Systematic Reviews and Meta-Analyses (PRISMA) (12) for the development of programs, data analysis, and reporting.

### 2.1 Search strategy

A systematic search of relevant publications was conducted in the following databases: Embase, PubMed, Cochrane Library, Wanfang database, and China National Knowledge Infrastructure (CNKI) from April 2013 to April 2023. We used a combination of keywords and Medical Subject Heading (MeSH) terms to represent the following concepts: [(cerebral embolic protection device) OR (cerebral embolic protection system)] AND [(transcatheter aortic valve implantation) OR (transcatheter aortic valve replacement)].

### 2.2 Study selection

#### 2.2.1 Inclusion criteria

This analysis exclusively comprised RCTs, which were required to meet two specific criteria: (1) comparative studies investigating stroke outcomes with or without CEPD during TAVI and (2) a minimum of 30 days of follow-up. The primary endpoint was stroke (not only cerebrovascular accidents but also death due to cerebrovascular accidents). Including stroke-related deaths in the analysis was important as they serve as an indication that the patient suffered from a stroke. Additional clinical outcomes included major bleeding, acute kidney injury, and major vascular complications.

#### 2.2.2 Exclusion criteria

The following are the exclusion criteria for the study: (1) studies that are not written in English or Chinese, (2) studies where the primary endpoint was not stroke, (3) studies with overlapping articles, replicated data, or replicated studies, and (4) studies where the valve model or CEPD was uncertain.

### 2.3 Data extraction

Two authors (CW and JH) extracted the data from the included studies. The extracted data included the study design, year of publication, number of patients, patient demographic characteristics, valve type, CEPD type, TAVI route, outcome definitions, and clinical outcomes (stroke). Any disputes could be resolved by a third author (LL) after discussions.

### 2.4 Quality assessment

The Cochrane collaboration's tool was used to assess the risk of bias in RCTs (13). Funnel plots were used to demonstrate the publication bias of the included RCTs (13).

### 2.5 Statistical analysis

The RevMan 5.4 software was used for this meta-analysis. The effect size was determined using 95% confidence intervals (95% CI) and risk ratios (RR). We used the *I*-squared (*I*^2^) statistic to evaluate the statistical heterogeneity. When *I*^2^ < 50%, the fixed-effects model will be used; otherwise, the random-effects model will be selected. Sensitivity analyses were performed by removing one study at a time. A significance level of *P* < 0.05 was used to determine statistical significance.

Mantel–Haenszel methods were used for this meta-analysis. These methods employed fixed-effect meta-analysis methods with different weighting schemes depending on which effect measure (e.g., risk ratio, odds ratio, risk difference) was used. They have been shown to have better statistical properties when there are few events (10).

## 3 Results

### 3.1 Search results and inclusion studies

**Figure 1:**
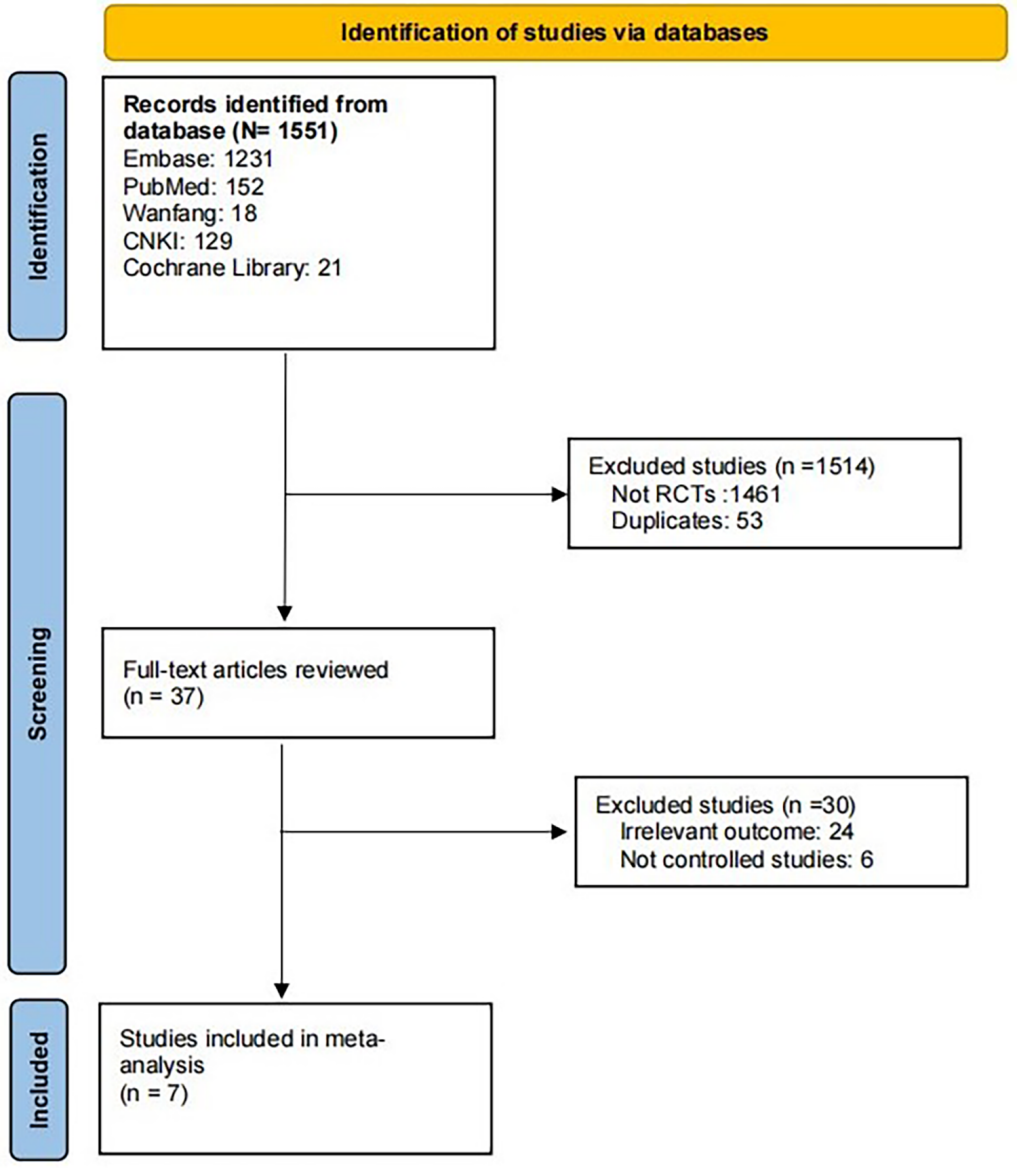
The inclusion and exclusion criteria of the study.

Through the above search method, a total of 1,551 publications were obtained. Upon thorough examination of the abstract and full text, seven articles (11, 14–19) with a total of 4,048 patients were included. The article screening process is shown in Figure 1.

### 3.2 Study characteristics and quality assessment

**Figure 2:**
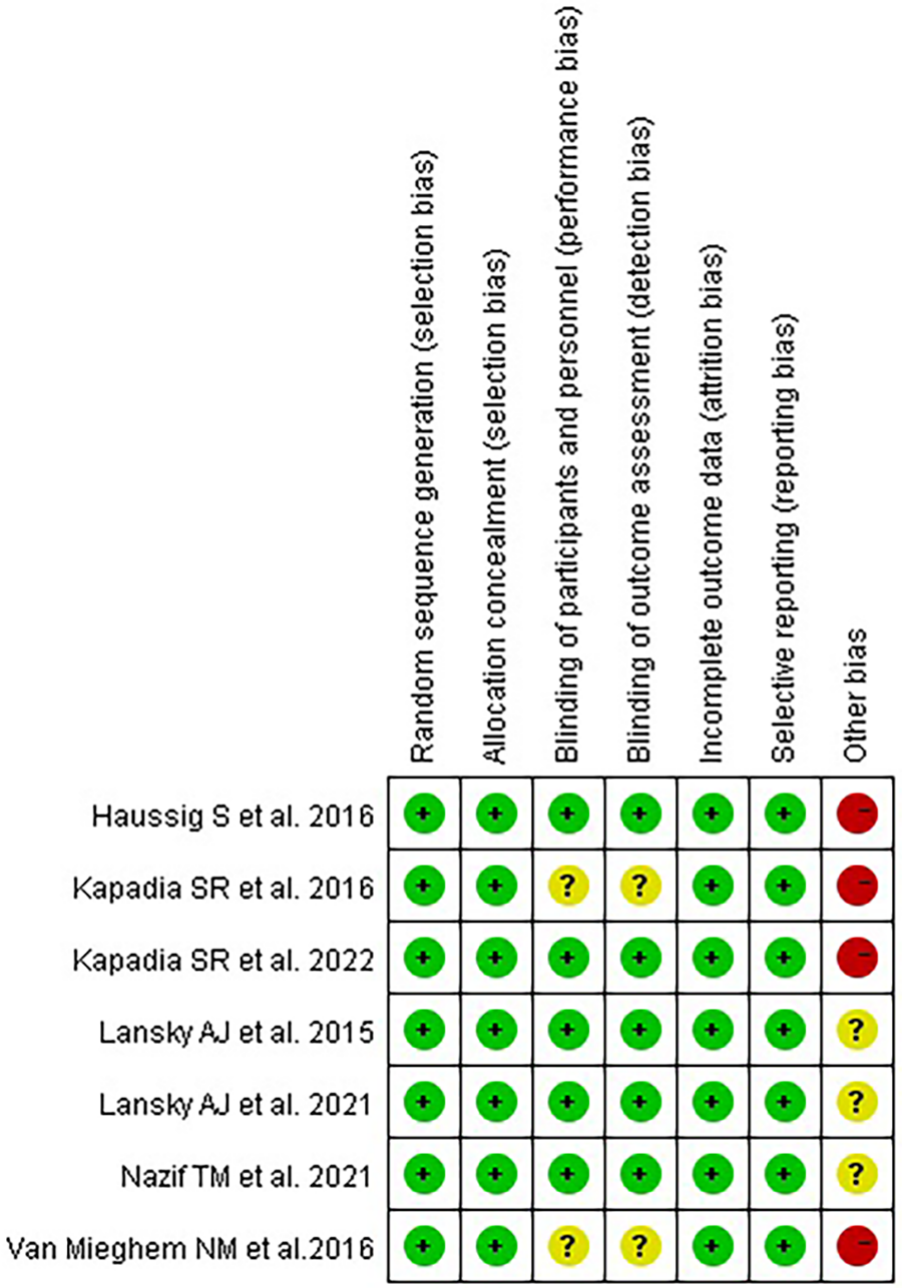
Quality assessment of RCTs.

The characteristics of the included studies are shown in Table 1, and the quality assessment is shown in Figure 2.

**Table 1:** Characteristics of the included studies.

Author & Year	Country	Male sex	Age	Valve type	CEPD^a^ type	TAVI^b^ route	Outcome definitions	Follow-up period	Multicenter	*N* (CEPD)	*N* (total)
Haussig et al. 2016 (14)	Germany	50.00%	79.6	CoreValve	TMCA^c^	Unclear	NIHSS^e^	30 days	No	50	100
Kapadia et al. 2016	USA	47.90%	83.4	Sapien XT-17.8% Sapien 3-52.4% CoreValve-3.9% CoreValve Evolut R-25.9%	I&LCCA^d^	Femoral access	MACCE^f^	30 days	No	121	363
Kapadia et al. 2022 (11)	USA	50.80%	78.9	Balloon-expandable valve-64.5% Balloon dilation-23.9% Native bicuspid valve-8.5% Bioprosthesis: nonnative valve-3.1%	I&LCCA	Femoral access	NIHSS	30 days	Yes	1,501	3,000
Lansky et al. 2021 (17)	USA	57.00%	80.5	CoreValve-33% Sapien 3-61% Others-6%	TMCA	Femoral access	NIHSS	90 days	Yes	46	85
Lansky et al. 2015 (16)	USA	45.90%	82.4	Sapien 3/XT-63.5% CoreValve-31% Others-3.5%	TMCA	Femoral access	MACCE	30 days	Yes	141	204
Nazif et al. 2021 (18)	USA	56.50%	78.6	Medtronic CoreValve-36.2% Edwards SAPIEN-61.6% Other-2.2%	TMCA	Femoral access	NIHSS	30 days	Yes	112	231
Van Mieghem et al. 2016 (19)	Netherlands	55.00%	81.5	Sapien 3-54% Medtronic CoreValve-25% Sapien XT-15% Balloon dilatation-5% Portico-1%	I&LCCA	Femoral access	NIHSS	30 days	No	32	65

^a^ CEPD: cerebral embolic protection device.

^b^ TAVI: transcatheter aortic valve implantation.

^c^ TMCA: most of them were TriGuard^TM^ CEPD, filter cover the three major cerebral arteries (innominate, left common carotid, and subclavian arteries).

^d^ I&LCCA: most of them were Sentinel^TM^ CEPD, filter cover the innominate and the left common carotid artery.

^e^ NIHSS: National Institute of Health Stroke Scale.

^f^ MACCE: major adverse cardiac and cerebrovascular events.

### 3.3 Outcomes

**Figure 3:**
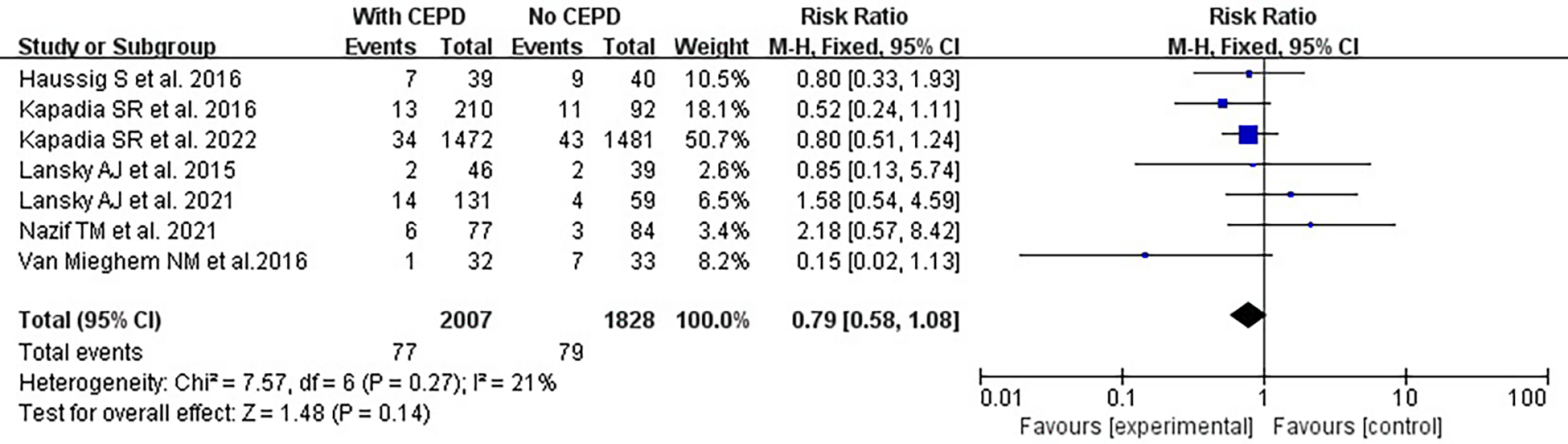
Effects of a 30-day stroke occurrence when comparing the use of CEPD and NOT during TAVI in RCTs. CEPD, cerebral embolic protection devices; RR, risk ratio; M–H, Mantel–Haenszel; TAVI, transcatheter aortic valve implantation.

**Figure 4:**
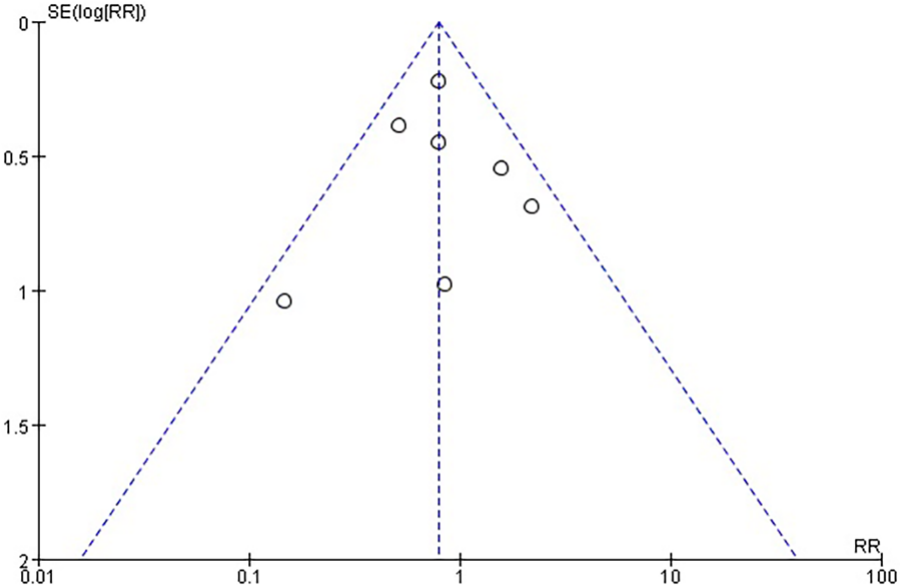
Publication bias.

There was no statistically significant difference in the risk of stroke within 30 days between the use of CEPD during TAVI and the control group: RR 0.79, 95% CI 0.58–1.08, *p* = 0.14, *I*^2 ^= 21% (Figure 3). A sensitivity analysis was conducted by systematically excluding one study at a time, and the results were not significantly changed (Table 2). An inspection of the funnel plot (Figure 4) showed no apparent asymmetry, indicating a possible absence of publication bias.

**Table 2:** Sensitivity analysis.

Exclude	RR/CI	*P*	*I* ^2^
Haussig et al. (14)	0.79 [0.57, 1.10]	0.16	34%
Kapadia et al. 2016 (15)	0.85 [0.61, 1.20]	0.36	18%
Kapadia et al. (11)	0.79 [0.51, 1.21]	0.28	34%
Lansky et al. (16)	0.79 [0.58, 1.08]	0.14	34%
Lansky et al. (17)	0.74 [0.53, 1.02]	0.07	15%
Nazif et al. (18)	0.74 [0.54, 1.02]	0.07	6%
Van Mieghem et al. (19)	0.85 [0.62, 1.16]	0.31	0%

**Figure 5:**
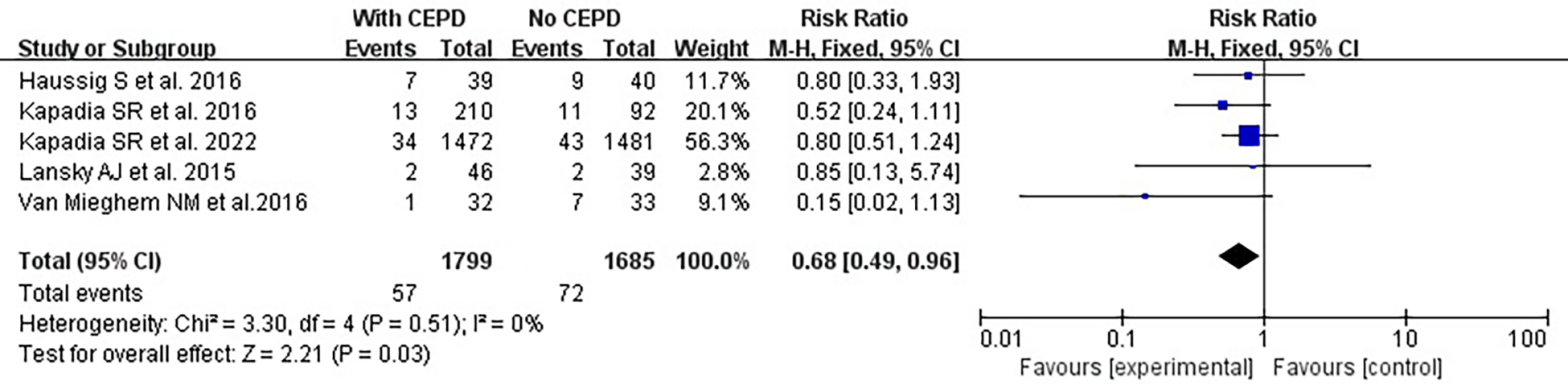
High-quality meta-analysis: effects of a 30-day stroke occurrence between using CEPD and NOT during TAVI in RCTs. CEPD, cerebral embolic protection devices; RR, risk ratio; M–H, Mantel–Haenszel; TAVI, transcatheter aortic valve implantation.

However, due to the misplacement of certain CEPDs or vascular damage observed in the studies conducted by Lansky et al. (17) and Nazif et al. (18), the efficacy of CEPD was decreased and the risk of stroke was increased. Consequently, a high-quality meta-analysis was performed after excluding the two RCTs mentioned above. The risk of stroke was lower in the CEPD group: RR 0.68, 95% CI 0.49–0.96, *p* = 0.03, *I*^2 ^= 0% (Figure 5).

**Figure 6:**
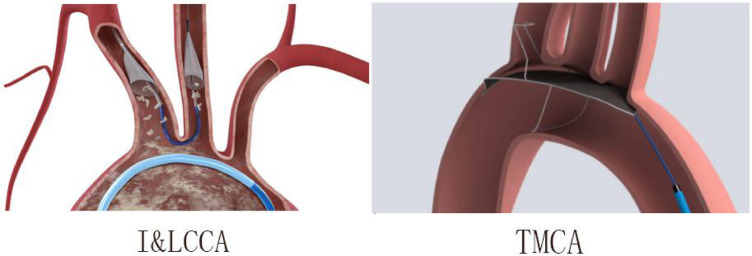
The innominate–left common carotid artery type (I&LCCA) and the three major cerebral arteries type (TMCA) in this meta-analysis.

**Figure 7:**
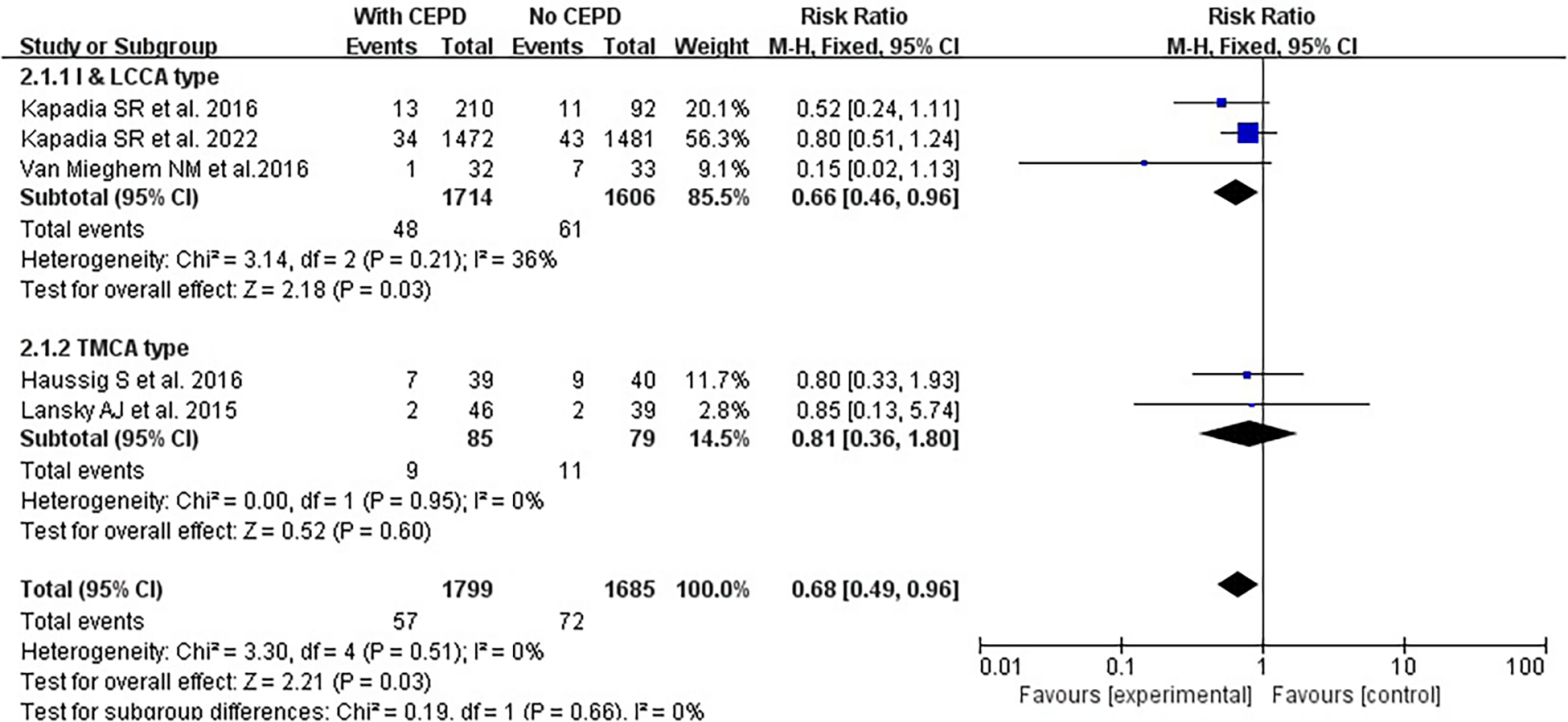
Subgroup analyses of the I&LCCA type and the TMCA type. I&LCCA: A type of CEPD that filter cover the innominate and the left common carotid artery; TMCA: A type of CEPD that filter cover the three major cerebral arteries (innominate, left common carotid, and subclavian arteries). CEPD, cerebral embolic protection devices; RR, risk ratio; M–H, Mantel–Haenszel; TAVI, transcatheter aortic valve implantation.

Based on the CEPD design types of the enrolled studies, there were two categories: (1) The I&LCCA group mostly utilized the Sentinel^TM^ CEPD, which covered the innominate and the left common carotid artery and (2) the TMCA group mainly used the TriGuard^TM^ CEPD, which covered the three major cerebral arteries (innominate, left common carotid, and subclavian arteries) (Figure 6). A subgroup analysis was conducted according to the type of CEPD. The risk of stroke was lower in the I&LCCA-type CEPD group: RR 0.66, 95% CI 0.49–0.96, *p* = 0.03, *I*^2 ^= 36%. However, there was no significant difference in the risk of stroke when using the TMCA-type CEPD group: RR 0.81, 95% CI 0.36–1.80, *p* = 0.60, *I*^2 ^= 0% (Figure 7).

## 4 Discussion

In this meta-analysis, we analyzed the incidence of stroke within 30 days after TAVI, and the risk of stroke was found to be lower in the CEPD group during TAVI. Furthermore, the subgroup analysis indicated that the I&LCCA-type CEPD could reduce the risk of stroke during TAVI, but the TMCA type could not.

**Figure 8:**
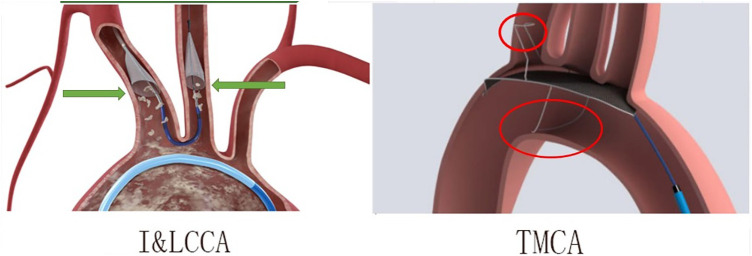
The differences between the I&LCCA type (the innominate–left common carotid artery type) and TMCA type (the three major cerebral arteries type). I&LCCA type: more suitable, less vascular endothelium damage; TMCA type: integrated design, exerting pressure on vascular endothelium.

After our discussion, the reasons were as follows:

1. The products design of the I&LCCA type consists of two independent funnel-shaped strainers that are designed to better align with vessels and be positioned more accurately. Moreover, the two funnels were fixed to the target vessel through blood flow pressure, resulting in less pressure and minimal damage to the vascular endothelium. However, the TMCA type was developed based on an integrated design, which may not be suitable for all sizes of blood vessels. It functions by exerting pressure on the vascular endothelium to maintain the desired position, although this can potentially result in the formation of blood clots (Figure 8).
2. TMCA is a new type of CEPD that may require further improvement in its design and further enhancement of operator proficiency.

Given the abundance of research demonstrating the safety of CEPD, safety analysis was not conducted in this study (17, 20–22).

### 4.1 Limitation

In addition, the outcomes of the included RCTs were influenced by the following factors: (1) Valve type: There were more than five types of valves. By utilizing only one type, the outcome may be improved. (2) Outcome definitions: Both the NIHSS and MACCE were used to assess the occurrence of stroke. It is recommended to use only one. (3) The RCTs conducted by Haussig et al. (14), Kapadia et al. (15), and Van Mieghem et al. (19) were not multicenter studies.

## 5 Conclusion

In conclusion, our meta-analysis revealed that the I&LCCA-type CEPD could reduce the risk of stroke within 30 days after TAVI, but the TMCA type could not. The efficacy of the TMCA-type CEPD might be demonstrated by the implementation of large-scale research with a substantial sample size, focusing on a single valve type and a single outcome definition. In addition, conducting multicenter studies and ensuring that operators are well trained would contribute to the validity of the findings. A new RCT (NCT05295628) is currently underway to investigate the efficacy of the I&LCCA-type and TMCA-type CEPD. The trial aims to enroll a total of 532 subjects undergoing TAVR at up to 30 investigational sites in the United States. The results of this trial may provide valuable insights into the true efficacy of the TMCA-type CEPD.

## Data Availability

The original contributions presented in the study are included in the article/Supplementary Material, further inquiries can be directed to the corresponding author.
